# Flexible Use of Urban Resources by the Yellow Mongoose *Cynictis penicillata*

**DOI:** 10.3390/ani9070447

**Published:** 2019-07-16

**Authors:** Nadine Elizabeth Cronk, Neville Pillay

**Affiliations:** School of Animal, Plant and Environmental Sciences, University of the Witwatersrand, Johannesburg, Private Bag 3, WITS 2050, South Africa

**Keywords:** anthropogenic food sources, diet, habitat, home range, urbanization, yellow mongoose

## Abstract

**Simple Summary:**

Many species have become locally extinct because of urbanization. However, many thrive in urban areas because they originally have, or they have acquired, features that enable them to exploit urban areas. We studied the ecology of the urban yellow mongoose, a recent urban dweller in parts of South Africa. We investigated the diet, space use and activity habits of yellow mongooses, and whether they exploit residential gardens. Similar to their non-urban counterparts, yellow mongooses in urban areas fed on insects, particularly in spring/summer. The presence of human food items, small mammals and birds in scats increased during autumn/winter, when insects are known to be less abundant. Camera trap footage revealed that, similar to their non-urban counterparts, yellow mongooses in urban areas were more prevalent in open habitats, and showed an early morning, late afternoon diurnal activity pattern. These urban mongooses were more frequently near human residences than at sites further away. Their home range size was considerably smaller than that of non-urban mongooses and overlapped more with human residents during autumn/winter than during spring/summer. Overall, the urban yellow mongooses displayed characteristics similar to non-urban mongooses, particularly in their diet, habitat use and activity patterns. Yet, they modified their diet by including human food, occurred in gardens, and had smaller home ranges, indicating modifications for urban life.

**Abstract:**

Several species are negatively impacted by urbanization, while others thrive in urban areas by exploiting anthropogenic habitats matching their pre-existing niche preferences, or by modifying their behavior for urban life. We studied the ecology of a recent urban resident, the yellow mongoose, in an urban ecological estate in South Africa. We assessed urban dwelling yellow mongooses’ diet, spatial and temporal occurrence, home range size, and whenever possible, compared our findings to the published literature on their non-urban conspecifics. Additionally, we evaluated occurrence overlap with residential gardens. Similar to their non-urban counterparts, scat analyses revealed that yellow mongooses in urban areas fed mainly on insects, particularly during spring/summer. In the colder months, anthropogenic items, small mammals and birds in scats increased. Camera trap surveys showed that the mongooses were common in open habitats, similar to previous studies, and exhibited a species-typical bimodal diurnal activity pattern. The occurrence of these mongooses was greater near human residences than at sites further away. Home range sizes were considerably smaller than those of non-urban mongoose. Mongoose occurred in residential gardens, more so during the colder months. The urban yellow mongooses’ diet, habitat preference and activity patterns were similar to non-urban conspecifics. Nonetheless, the exploitation of anthropogenic food sources, occurrence in residential gardens and smaller home range sizes showed that they respond flexibly to urbanization, and these modifications might aid in their success in urban areas.

## 1. Introduction

Habitat fragmentation and the loss of natural habitats is a leading cause of the decline in wildlife populations worldwide [1,2]. Urbanization is a primary factor contributing to this fragmentation, and severely altered environmental conditions are largely attributed to the increasing human population and concurrent urban expansion [3,4]. However, urban areas, including large cities, provide an overabundance of anthropogenic resources, as well as a concurrent reduction in natural predators [5,6], potentially providing shelter for urban wildlife. As a result, several studies have shown how species prosper in urban areas, particularly small to medium sized meso-carnivores [4,7,8,9,10]. These studies have shown how different aspects of animal biology are modified, such as being flexible in diet, activity and space use, aiding in the success of some wildlife in urban environments. Nevertheless, few have considered a multi-variable assessment of an urban species’ biology.

Urbanization affects carnivores in several ways. The diets of urbanized carnivores typically include a combination of vertebrate and invertebrate prey, and often plant material, and they also tend to exploit available anthropogenic resources [7,9,10]. For example, in the red fox *Vulpes vulpes*, anthropogenic items dominate stomach contents, particularly in individuals sampled closer to city centers [11]. Similarly, scats of martens *Martes spp*. in urban areas contain a high proportion of human garbage, while martens in non-urban areas feed mainly on insects [8]. Habitat use of animals inhabiting urban areas is concurrently also largely influenced by the abundance and availability of preferred food resources [9,12]. An animal’s home range size is affected by population density, and larger home range sizes are generally correlated with smaller population densities [3]. In urban areas, with the increased resource availability and reduced predation, species are able to attain higher population densities [1,6]; numerous studies have shown that home range sizes of animals are greatly reduced in urban areas, largely attributed to both the increase in population densities and greater resource availability [4,13,14]. Urbanization also impacts activity patterns, particularly in larger carnivores, as a mechanism of temporal avoidance of conflict with humans [9]. For example, Beckman et al. [15] showed that black bears *Ursus americanus* in urban areas were less active and changed to being nocturnal; both these behaviors were attributed to available anthropogenic food sources and direct human disturbances. Similarly, tigers *Panthera tigris* exhibited lower activity levels in areas where they overlapped spatially with humans [16]. 

Urban expansion is inexorable, and we can predict an ever-increasing number of wild animal occurrences in urban areas worldwide. It is, therefore, crucial to understand the ecology of urban species, particularly with the goal of introducing effective management plans should the need arise [4,13]. Our focus here is on the yellow mongoose *Cynictis penicillata*, a small carnivore widely distributed in southern Africa, most commonly in open grassland habitats. Free-living yellow mongooses in southern Africa have been extensively studied, including many aspects of their ecology. They are diurnal and largely solitary foraging insectivores, but also commonly consume small mammal and avian prey. The species lives in groups of 2–13 individuals, and has evolved cooperative breeding. Mating begins in July, with a gestation period of approximately two months. Home range size is estimated as a mean of 70 ha (male home ranges being typically larger than females), and is dependent on population density of the study areas [17,18,19,20,21]. Reported sightings in close proximity to urban areas date back to 1999 [22], and the yellow mongoose is now a common urban resident, but research into the ecology of urban populations is a recent development [23,24].

We investigated the diet, spatial occurrence, activity patterns, and home range of the yellow mongooses in urban Johannesburg (South Africa), and compared these results to those of mongooses in non-urban environments in the literature. The aims of our study were to (1) describe how yellow mongooses exploit an urban environment; and (2) establish whether the yellow mongooses have a different eco-type in urban than non-urban environments. Our multi-pronged approach allowed us to consider whether one or several ecological characteristics were modified because of urbanization, and whether certain characteristics were retained from a non-urban existence, potentially making them pre-adapted to an urban setting. Overall, we expected that the yellow mongooses have modified their diet, space use and activity by incorporating available anthropogenic resources to exploit urban areas. The yellow mongooses in non-urban areas show strong seasonality in their diet as a response to food availability. We tested whether such seasonality is dampened in urban areas, where resources (e.g., food, shelter) are more predictable and constant all year round. We also investigated whether this seasonality is also dampened in space use and activity, factors that have not been studied in non-urban yellow mongooses.

## 2. Materials and Methods

### 2.1. Study Site

This study took place from March 2015 to April 2018. Field work took place in an urban ecological estate, Meyersdal, Johannesburg, South Africa (26°17′10.4″S 28°05′14.7″E). The climate in the area is warm and temperate, with an average annual temperature of 16 °C, and average precipitation of 723 mm. The warmest temperatures occur in December–February (average minimum of 14 °C and average maximum of 26 °C) and the coldest in July (average minimum of 3 °C and average maximum of 19 °C). The estate (480 ha) consists of residential development interspersed with natural areas dominated by grassland vegetation, with dispersed areas of tree cover and undulating rocky hills [25]. Numerous outdoor trails for recreational use are located throughout the entire estate, and wildlife also occur in close proximity to human residences and utilize gardens and corridors between residential houses. Several small to large sized mammal game species occur here, as well as numerous smaller mammals, such as rock hyraxes *Procavia capensis,* porcupines *Hystrix africaeaustralis* and spotted genets *Genetta genetta*. Potential competitors of yellow mongooses include feral and domestic cats *Felis catus*, slender mongooses *Galerella sanguinea* and black backed jackals *Canis mesomelas*, although yellow mongooses are the most abundant carnivore (estimated at 30–40 individuals per 100 ha; pers. obs.). Predators of the yellow mongooses are mostly jackals and a breeding pair of black eagles *Ictinaetus malaiensis*. Yellow mongooses are also found in neighboring Johannesburg south twons, such as Rosettenville and Alberton, which surround Meyersdal. 

### 2.2. Diet

Yellow mongoose diet was assessed through scat sample analysis. Fresh scat samples were collected from 10 sampling sites (Figure S1), once a month, from March 2015 to March 2016. Total sample size was 1300 scats collected over the entire study period (13 collection periods). Yellow mongoose scats were identified on the basis of their odor and appearance and were easily located at middens near denning sites, characterized by underground dens in the open grassland or in rock crevices above- and belowground. Scat samples were morphologically analyzed using the protocol described by Cronk and Pillay [24]. The contents of scats were categorized into five types: plants, invertebrates (insects), mammals, birds, and anthropogenic. Previous studies have reported that yellow mongooses feed on amphibians and reptiles, but none were identified in scat samples in this study. To identify common prey items, a sub-sample of 200 scat samples was further analyzed and remains of invertebrates, mammalian and avian prey taxa were morphologically identified to appropriate taxonomic levels (order and family) using reference catalogues, and the assistance of relevant specialists at the University of the Witwatersrand. 

The prevalence and relative importance of food categories in the diet were quantified by using two index methods: (1) frequency of occurrence (F.O.), calculated by dividing the total frequency of a food category by the total number of scats analyzed, and expressed as a percentage; and (2) percent occurrence (P.O.), calculated by dividing the occurrence of a food category by the total number of occurrences of all food categories, and expressed as a percentage [26]. F.O. and P.O. were used since both use the presence, rather than the volume, of diet items to compare items of varying levels of digestibility (e.g., bones versus soft human food; [9]). Moreover, these two methods have been used in previous studies, allowing for inter-study comparisons [24]. Plants present in scats were dominated by grasses and leaves. These items do not form part of the yellow mongooses’ diet, and their presence in the scat was attributed to indirect ingestion when consuming other food [17]. Therefore, plants were excluded from the calculation of percent occurrence to avoid overrepresentation of their importance in the yellow mongoose’s diet.

### 2.3. Spatial Occurrence and Activity Patterns

We used camera trap surveillance to assess the spatial and temporal occurrence of yellow mongooses throughout the estate. Three motion triggered Bushnell Essential^®^ camera traps were used over the sampling period of May 2015 to March 2016. These were attached to rigid surfaces (rocks/trees/poles) and angled to maximize field of view of wildlife trails. Camera traps were active 24 h a day, and remained at a site for a 2-week period before being moved to a new site approximately 200 m away; no bait was used and camera traps were not placed at known mongoose dens. Camera traps were never placed in the same area in a season but were sometimes placed in close proximity to previous sites in different seasons. Cameras were checked once a week to change batteries and memory cards.

A total of 66 sites were sampled throughout the study period (Figure S1). At each site, we recorded the GPS coordinates, the relative distance to human residences (in meters), and the vegetation cover (open: areas dominated by open fields of grasses; or closed: areas dominated by tree cover or dense bushes); these variables aided in identifying relative use of residential areas (as a proxy for access to and potential use of anthropogenic resources) as well as micro-habitat type use. From photographs obtained where a yellow mongoose was present, we recorded the date and time of occurrence; consecutive captures of yellow mongooses within a 10 min time frame were considered as a single occurrence (i.e., the same individual; [27]). Time stamp data were used to generate the yellow mongooses’ activity patterns. 

### 2.4. Home Range and Residential Overlap

Trapping, collaring, and tracking of two male and two female mongooses took place from March 2016 to May 2017. We used single door humane animal traps (80 × 29 × 33 cm galvanized wire mesh) placed at four different sites in different regions of the estate (at least 1 km apart), in an attempt to avoid capturing individuals that were likely to congregate in social groups (resulting in spatial correlation between individual mongooses). Traps were camouflaged, baited with off-cut deli meats, and set out in the early morning before mongoose activity was expected to start. Traps were checked every hour to reduce the stress caused by captivity. Trapped yellow mongoose were immobilized using a 0.06 mL/kg Medatomidine and 6 mg/kg Ketamine, which was administered intramuscularly by a registered veterinarian. We recorded standard weights and body measurements before fitting a GPS collar. Collars were manufactured by Africa Wildlife Tracking (HAWK-UHF device, AWT CC, Pretoria, South Africa), weighed between 65 and 85 g, and were designed with an easy release point that required minimal stress to break when snagged (i.e., repetitive pulling by mongoose would break the collar). To maximize battery life, collars were programmed to record GPS coordinates (hereafter referred to as fixes) every 3 h during daylight hours (between 5 a.m. and 7 p.m.), to coincide with yellow mongoose activity; the 3 h interval accounted for potential spatial auto-correlation between fixes. Once the collar was fitted, the mongoose was returned to the trap, and an anesthetic reversal of 0.3 mg/kg Atipamezole was administered. The veterinarian monitored when the mongoose was mobile again in order to assess whether there was any discomfort or distress from the collar. The collared mongoose was subsequently released at the site of capture. 

To collect data from the collars, collared individuals were located by means of the triangulation method, using a hand-held H antenna (AWT CC, 433 MHz), and a portable VHF receiver (HAWK 433 MHz transceiver, AWT CC). Each collar had a reception range of ±1 km, and data could be downloaded from within a ±50 m range of a collared mongoose. Tracking took place on foot. All GPS locations were automatically stored on the collar which required a single download from the receiver every 2 weeks. The receiver was connected to a computer and GPS coordinates were then downloaded for later analysis. 

Home range size was defined by the area navigated by each collared mongoose while engaging in different activities. Home range size was established using minimum convex polygon (MCP) and kernel density estimates (KDE) methods for comparison with previous studies. Furthermore, GPS data were plotted to establish occurrence of mongooses within residential gardens, as a proxy for potential access to, and use of human provided food resources. We assessed the overlap with human residences, defined as the percentage of GPS locations found within residential gardens [7]. 

### 2.5. Data Analyses

All data were recorded in, and graphs generated, using Microsoft Excel^®^ (Microsoft Corporation, 2007). All statistical analyses were conducted using R Statistical Software (www.r-project.org, R version 3.4.3). For diet and spatial occurrence data analyses, sampling periods were divided into four seasons according to South Africa’s weather patterns: Colder seasons—autumn (March–May) and winter (June–August); and warmer seasons—spring (September–November) and summer (December–February).

#### 2.5.1. Diet

A generalized linear model (coded glm), with a Poisson distribution and log link function, was used to identify the significant predictors of percent occurrence (dependent variable). The model comprised of single and first order interaction effects between food category and season as fixed effects. We used the lsmeans function in R to perform post-hoc tests to interpret significant outcomes. Additionally, we illustrated seasonal variation graphically by comparing percent occurrence of each food category per month.

#### 2.5.2. Spatial Occurrence and Activity Patterns

We recorded the total number of occurrences of yellow mongoose at each site. Using a generalized linear model (coded glm), we analyzed whether the fixed effects—(1) distance relative to residences (in meters; as a continuous predictor), (2) cover (open/closed) and (3) season (autumn/winter/spring/summer)—had a significant effect on the number of photographs captured of yellow mongoose (dependent variable). We used the lsmeans function in R to perform Tukey post-hoc tests to interpret significant fixed effects. Furthermore, activity profiles comparing the colder (autumn and winter) and warmer (spring and summer) seasons were generated using time stamp data from all photographs obtained. To visualize the data graphically, we used the R package ‘*overlap*’ and the function ‘*overlapEst*’ (type = Dhat4, smoothing constant adjust = 1) to obtain pair-wise temporal overlap coefficients (Δ; [28,29]), in order to identify seasonal variation in activity patterns. Temporal overlap of activity patterns between cold and warm seasons was estimated using kernel density estimation in R following the methods proposed by Ridout and Linkie [29] (where c = 1). The overlap coefficient generated (Δ) ranges from 0, indicating no overlap, to 1, indicating complete overlap [30].

#### 2.5.3. Home Range and Residential Overlap

All GPS fixes (location error estimate of 2–3 m) were downloaded from the receiver and recorded on a spreadsheet. We established the home range size for each mongoose by calculating 50%, 95% and 100% isopleths, according to MCP and KDE (h = href) methods in R using the package adehabitatHR [31]. For the 100% KDE, the model did not converge and, subsequently, unrealistically large home range sizes were generated; these were not included in analyses (see [32]). A minimum of 50 GPS fixes are required to estimate the home range size of yellow mongooses [20]. However, Balmforth [32] reported that home range sizes stabilise within the range of 100–250 GPS fixes, which was achieved for each mongoose in this study for the estimation of overall home range size. Because of the small number of mongooses collared, we qualitatively compared home range sizes between female and male mongooses. We descriptively compared home range sizes by season sampled for each mongoose separately by grouping the months sampled for each individual into seasons. In addition, overlap with human residences (as a percentage of GPS locations found within residential gardens) was compared between seasons to establish variation in use of residential gardens per individual mongoose. Kernel density estimate home range images (showing utilization distribution of the 50% and 90% isopleths) were generated using R, and graphically represented using Quantum GIS (QGIS 3.2 Bonn; http://www.qgis.org).

## 3. Results 

### 3.1. Diet

An analysis of the 1300 scat samples indicated a variety of food items present in the yellow mongoose diet (Table 1). Invertebrates ranked first as the most frequently occurring components in the scat, followed by anthropogenic items, mammals and then birds, according to two methods of analysis (Table 1). Plant material (mostly grass) was present in a large proportion of scat samples, but we attributed this to indirect ingestion. The sub-sample analysis to identify components of each prey food category identified Isoptera (termites; 70.1%) and Orthoptera (grasshoppers and crickets; 53.5%), Rodentia (mice and rats, such as *Rhabdomys* spp. and *Otomys irroratus*; 82.5%) and Columbidae (doves and pigeons; 85.7%) as the main insect, mammal and bird taxa present in scats, respectively (Table S1). Anthropogenic items included garbage (foil, hard and soft plastics) and chicken eggs shells. Results of the generalized linear model indicated that percent occurrence was significantly affected by food category (χ^2^_3_ = 328.54, *p* < 0.001) and the food category × season interaction (χ^2^_9_ = 25.76, *p* = 0.002); season alone was not a significant predictor of percent occurrence (χ^2^_3_ = 0.23, *p* = 0.972).

**Figure 1:**
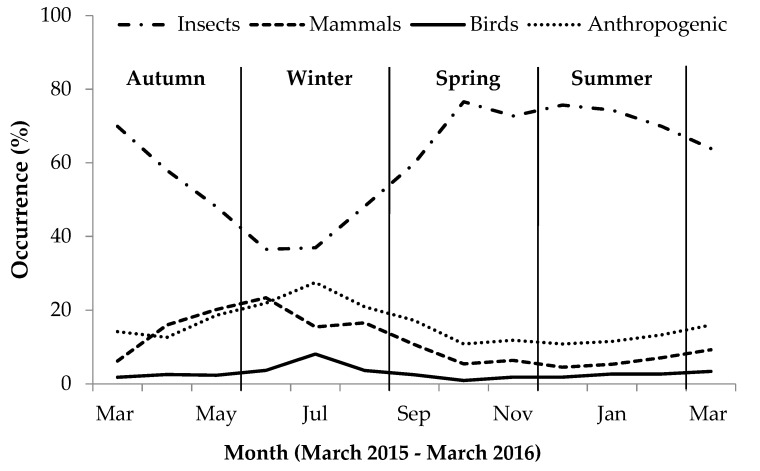
Monthly percent occurrence (%) expressing seasonal variation in occurrence of different food categories in yellow mongoose scat in the Meyersdal estate.

During autumn, invertebrate presence in scat gradually decreased while bird, mammal and anthropogenic item presence increased (Figure 1). During winter, bird and anthropogenic item occurrence in scat increased, while invertebrate and mammals prey occurrence decreased. During spring and summer, when temperatures increased, the occurrence of birds, mammals and anthropogenic items in scats gradually decreased while invertebrate occurrence increased considerably (Figure 1). The greatest seasonal variation was apparent in the occurrence of anthropogenic items and invertebrates in the scat: During winter, the occurrence of insect material in scats greatly decreased, while concurrent use of anthropogenic items increased during this period, whereas during summer, insect presence in scats was at its greatest and anthropogenic item occurrence was lower than during winter (Table S2). 

### 3.2. Spatial Occurrence and Activity Patterns

A total of 1289 independent photographs of yellow mongoose were captured by the camera traps (Table 2). The mean number of photographs (±SE) captured per site was 20 ± 3, and the mean distance from human residences for the camera trap sites was 357.57 m ± 38.4 m, with the closest site at 11.08 m and the furthest site at 1073.63 m (Table S3). Results of the statistical analyses indicated that distance from human residences (χ^2^_1_ = 35.17, *p* < 0.001) and cover (χ^2^_1_ = 15.12, *p* < 0.001) were significant predictors of the number of photographs captured; season was not a significant predictor (χ^2^_3_ = 1.91, *p* = 0.594). The overall model fit was F_(1,64)_ = 22.87, *p* < 0.001, with an R^2^ of 0.54 (adjusted R^2^ = 0.50). Post-hoc tests revealed that more photographs of yellow mongoose were captured at distances closer to human residences (*p* < 0.001), and yellow mongoose occurred significantly more at sites where the vegetation cover was classified as open vegetation than at sites classified as closed vegetation cover (*p* < 0.001; Figure S2). 

**Figure 2:**
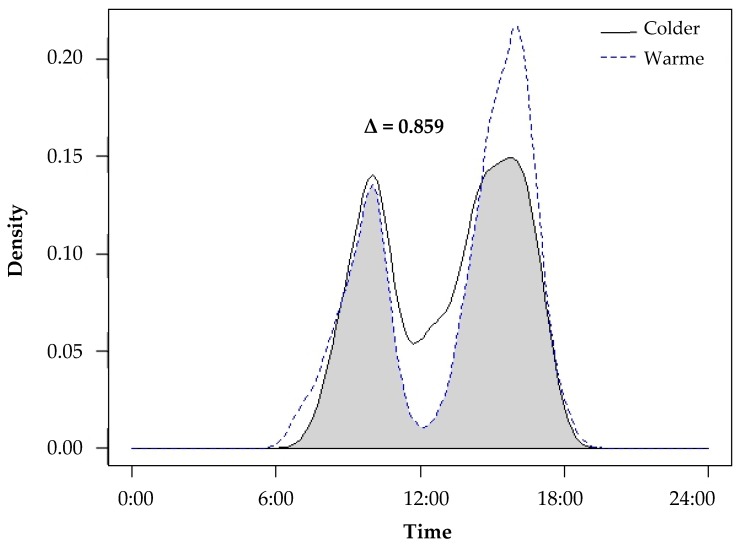
The activity patterns of yellow mongooses during the colder seasons (autumn and winter; solid line) and warmer seasons (spring and summer; dotted line) in the Meyersdal estate. The overlap coefficient between the periods (Δ) equals the area below both curves shaded grey in this figure, expressing seasonal overlap and variation in activity patterns.

Temporal data collected from camera traps revealed that yellow mongooses were primarily diurnal, with a mean start and end active time of 06:29 ± 00:28 and 18:19 ± 00:37 (±SE). Mongooses also exhibited bimodal peaks in activity, first between 10:00 and 11:00 and second between 16:00 and 17:00 (Figure 2). We compared the activity patterns between colder and warmer seasons (Figure 2) and found a high overlap coefficient (Δ = 0.859). The activity patterns were largely similar between the two periods, and mongooses maintained similar bimodal peaks in activity with slight variation in the start, end and peak activity times. During the warmer seasons (characterized by an earlier sunrise and later sunset), mongooses were active from earlier in the morning (as early as 06:28 a.m. as opposed to 07:40 a.m. during the colder seasons), and there was a decrease in activity during midday (when temperatures reached their maximum) and an increase in activity in the later afternoon/early evening (when temperatures are cooler). During the colder seasons (later sunrise and earlier sunset), activity was greater during midday and lower later in the afternoon.

### 3.3. Home Range and Residential Overlap

**Figure 3:**
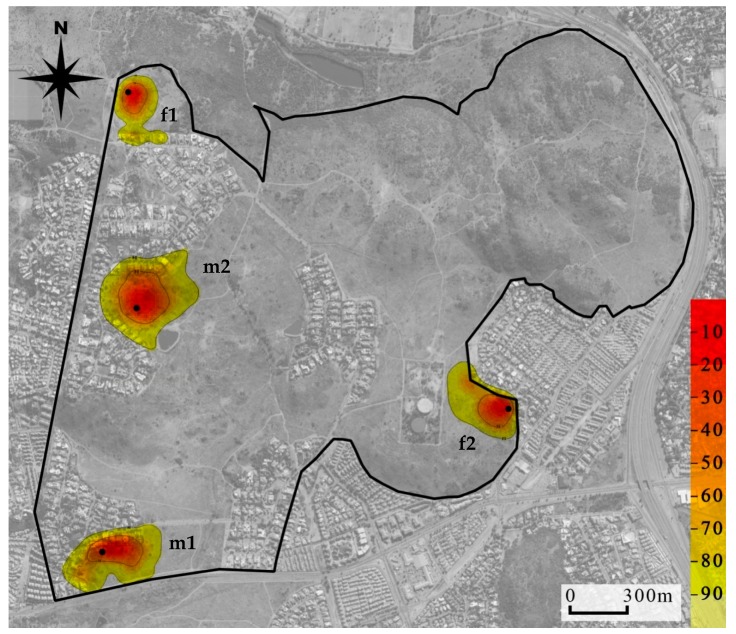
Location utilization density (by the kernel method) of four yellow mongooses overlaid on an aerial photograph of the Meyersdal estate. More red shows core use areas, while the more yellow shows wider area of use and extends to the 95% home range use; black dots indicate den location.

Mean (±SE) tracking duration of the four collared mongooses was 134 (13.31) days and the mean number of GPS fixes recorded was 385 (47.41; Table 3). The mean (±SE) home range size of the males was 14.61 ha (±4.48) and 13.37 ha (±4.61) and of females was 7.16 ha (±0.26) and 6.50 ha (±0.27), for MCP 95% and KDE 95%, respectively. Male home range sizes were generally larger than female home range sizes according to both estimates at all isopleth levels (Table S4). Mapping of home ranges indicated there was no overlap between these collared individuals (Figure 3).

The seasonal home range sizes showed similar patterns for both estimates; the smallest home range sizes occurred in spring, and the largest during autumn for the two females, and the smallest during winter and the largest during summer for the males (Table S5). Statistical analysis of seasonal home range size could not be done due to the limitation in data collected for each individual (home range size of each mongoose was not sampled in all seasons), and must be considered with some circumspection. 

**Figure 4:**
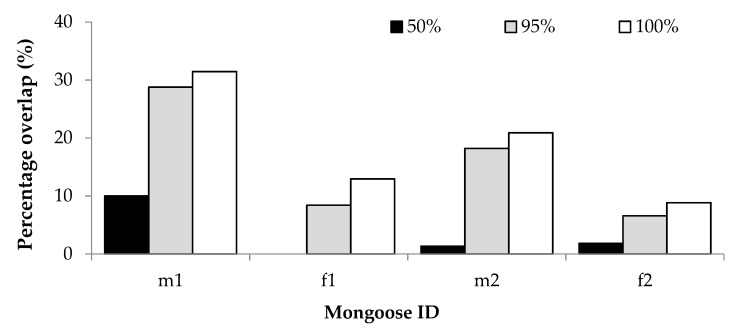
Percentage GPS location overlap with human residential gardens of two male (m1, m2) and two female (f1, f2) yellow mongooses for different home range isopleth levels in the Meyersdal estate.

For the overlap with human residences, percentage GPS overlap was proportionately similar for all mongooses; overlap was lowest at the 50% isopleth and greatest at the 100% isopleth (Figure 4). The two males had greater overlap with human residences, which could be attributed to their larger home range sizes. As a percentage of GPS locations recorded for each individual, the average percentage overlap with residential gardens at each isopleth level was 3.31% (50%), 15.49% (95%) and 18.53 (100%). For two individuals, home range overlap with residential gardens appeared to be greater during winter than during autumn, and for one individual the overlap was greater in autumn than in spring and summer (Table S6). 

## 4. Discussion

We investigated the ecology of urban-living yellow mongooses. We analyzed the main components of their diet, micro-habitat type use, activity patterns, home range size and overlap with human residences, as well as compared how some of these variables varied seasonally. No such studies have previously been conducted on other mongoose species in an urban setting.

Scat analysis of these urban yellow mongooses showed diets similar to previous studies on non-urban yellow mongooses, with insect (being the most prevalent), mammal, and bird prey present [17]. In the urban yellow mongoose scat, anthropogenic items occurred more than other food categories after insects. As reported previously in studies of non-urban yellow mongoose (e.g., [18]), their diet also varied seasonally, particularly for the invertebrates and anthropogenic item food categories, where insect presence was greater in summer than in winter and anthropogenic items presence was greater in winter than in summer. We observed that, as temperatures decreased, mammal and avian occurrences in scat increased while insects decreased, and anthropogenic items increased more than mammals and birds during this period. These results emphasize the importance of human foods in yellow mongoose diet in the study area, particularly during less favorable cold periods, when the availability of their main food (i.e., insects) is reduced [18]. Unfortunately, in dietary analysis of urban animals, only indigestible items are identified, and any other items that are easily digested (e.g., bread) are not normally accounted for [33]. Since we were able to identify only human garbage in scat, we propose the use of human food may be underrepresented in our study. 

In the urban estate, yellow mongooses occurred more often at sites with open vegetation cover, similar to their non-urban counterparts [21]; we did not find any seasonal influences on spatial occurrence. Habitat selection and use by carnivores is largely influenced by the abundance and availability of food resources [12]. For yellow mongooses, open habitat represents favorable conditions for obtaining insects, its main prey, which are most abundant in open grassland areas [18,19]. Animals utilizing open habitats should be better able to detect potential risk, and therefore in urban areas (typically considered to have reduced vegetation cover), these species may have a higher tolerance for human disturbance and may habituate to humans [34], which may also be the case here. In particular, yellow mongooses in our study area tolerate humans [23]. The number of photographs captured of yellow mongooses was far greater at sites located closer to human residences in the estate. Similarly, stone martens have greater occurrence closer to urban centers (i.e., a greater human presence) and also have a greater occurrence of anthropogenic items in their diets [7,8]. Therefore, we suggest that meso-carnivores that occur near to and tolerate human residences have access to anthropogenic food items.

Urban carnivores, typically medium–large carnivores, show shifts in activity patterns, which is largely attributed to temporal avoidance of humans [1,9,15]. In contrast, the yellow mongooses in our study maintained the same diurnal activity patterns, including seasonal variation in active periods, to those of their non-urban counterparts [21,35]. We suggest three reasons for this similarity. (1) The yellow mongooses’ tolerance for humans (without any negative consequences) might encourage their use of human residential spaces in daylight. (2) It is possible that the activity of the mongooses’ primary prey, invertebrates, shape their activity profile. (3). It is also likely that the smaller carnivores in urban areas are not persecuted or are more cryptic and hence retain their diurnal behavior whereas larger carnivores, which can pose a threat to humans, change their activity pattern to avoid persecution.

We investigated the home ranges of radio-collared individuals using two methods of analysis: MCP and KDE. In comparison to previous studies (focusing specifically on previously reported MCP 95%, 100% and KDE 95%), these analyses showed that the home range sizes of yellow mongooses in urban areas (average of 11.92 ha) was greatly reduced compared to their non-urban counterparts from studies using similar methods (average home range size of 38.41 ha; [21,32,35,36]). Numerous studies have reported reduced home range sizes of carnivores in urban areas, attributed to the increase in both population density and resource availability (e.g., [4,13,14]). The population density of yellow mongooses in the estate (30–40/100 ha) was greater than those reported in the non-urban populations in previous studies (for example, 6–7/100 ha reported by [35]; and 4–14/100 ha reported by [21,36]). We do not know whether the reduced home range sizes of yellow mongooses in our study (approximately 69% reduction in home range size) was the result of increased population density and/or increased resource availability, and requires further study. Similar to these previous studies, home range sizes of the two males were larger than those of the two females. Overall, home range sizes were smaller during the breeding period (winter and spring) than in the non-breeding period (summer and autumn; [21,32]). Seasonal variation in overall home range size of non-urban yellow mongooses has not been described, and was therefore not considered in this study.

The home ranges of the yellow mongooses overlapped with residential gardens. Their occurrence in residential gardens is likely due to habituation people and also the ease of access to anthropogenic resources (such as bird feeders, garbage bins and pet food; [1]). While not statistically assessed, the overlap with residential gardens was greatest during colder seasons. This would be expected, since urban animals are reported to supplement their diet in winter with anthropogenically provided foods [1], which are more likely to be found within residential gardens. 

## 5. Conclusions

Yellow mongooses are recent inhabitants of urban areas in South Africa due to rapid urbanization in the country. We used a multi-pronged approach to provide a comprehensive assessment of the mechanisms the species uses to exploit urban areas. The yellow mongooses demonstrated largely similar characteristics of diet, habitat use and activity patterns to that of their non-urban counterparts. Yet, they clearly exploited anthropogenic resources, which likely represents habituation and acclimatization to urban areas, and showed tolerance of human residents, use of residential gardens, and overall smaller home range sizes, which generally suggests behavioral modifications. Thus, it appears that they have capitalized on their pre-existing adaptations to exploit urban areas and have responded flexibly to urbanization, providing evidence of behavioral adaptation to an urban environment. We do not know whether the changes between urban and non-urban mongoose are due to isolation and genetic change, or simply because of habituation to people. The results of this study warrant further investigation of yellow mongooses in other urban areas, including an assessment of other environmental parameters (e.g., resource availability and distribution) for a better understanding of the habits of this urban meso-carnivore. 

## Figures and Tables

**Table 1:** Number of occurrences (*n*), frequency of occurrence (F.O.), percent occurrence (P.O.) and rank for each food category present in yellow mongoose scat (*n* = 1300); rank 1 being the highest ranking and 5 being the lowest.

Food Category	*n*	F.O.	Rank	P.O.	Rank
Invertebrates	943	72.54	1	65.62	1
Anthropogenic	260	20.00	3	18.09	2
Mammals	186	14.31	4	12.94	3
Birds	48	3.69	5	3.34	4
Plant material	731	56.23	2	*	*

* = percent occurrence for plant material was excluded to prevent overrepresentation of its importance in mongoose diet.

**Table 2:** Number of independent photographs of yellow mongooses in open and closed cover categories (*n* = number of sites for each cover category) during different seasons in the Meyersdal estate.

Season	Category of Cover		Total
	Open (*n* = 32)	Closed (*n* = 34)	
Autumn	205 (5)	8 (4)	213
Winter	248 (8)	105 (10)	353
Spring	279 (9)	77 (12)	356
Summer	297 (10)	70 (8)	367
**Total**	**1029**	**260**	**1289**

**Table 3:** Home range sizes from MCP and KDE estimates of individual male (m) and female (f) yellow mongoose (mass in grams), and tracking duration, in days, in the Meyersdal estate.

Mongoose Identity	Capture Date	Tracking Duration (no. Fixes)	Home Range Size (ha)				
			MCP			KDE	
			50%	95%	100%	50%	95%
m1 (750)	20-Mar-16	110 (299)	2.77	10.13	16.17	2.32	8.76
f1 (892)	20-Mar-16	113 (309)	1.63	7.42	8.93	1.43	6.76
m2 (768)	4-Sept-16	152 (445)	5.99	19.09	26.09	4.76	17.98
f2 (686)	20-Nov-16	162 (486)	1.81	6.9	8.57	1.69	6.23

## Supplementary Materials

The following are available online at https://www.mdpi.com/2076-2615/9/7/447/s1, Figure S1: Aerial map of the Meyersdal estate showing the 10 scat sample collection sites (triangles) and the 66 camera trap sites (diamonds); human residences within the estate are highlighted in yellow, Figure S2: The number of photographs of yellow mongooses captured on camera traps at different distances from human residences in open or closed vegetation cover in the Meyersdal estate, Table S1: Frequency of occurrence of various animal remains, identified to appropriate taxonomic levels (order and family), present in a subsample of scats (*n* = 200) of yellow mongooses in the Meyersdal estate, Table S2: Outcomes of the pair-wise comparisons for the lsmeans of the season and food categories (lsmeans post-hoc analysis output from R statistics), Table S3: Camera trap site information for the 66 sites sampled within the Meyersdal estate, Table S4: Comparison of male and female yellow mongoose home range sizes (mean ±SE; range), based on two estimates (MCP and KDE) for the 50%, 95% and 100% isopleth level in the Meyersdal estate. No estimates were possible for the KDE 100% isopleth, Table S5: Seasonal home range sizes, based on two estimates (MCP and KDE), of female (f) and male (m) yellow mongooses in the Meyersdal estate, Table S6: Seasonal percentage of total GPS locations (*n* = number of GPS locations) overlapping with human residential gardens, of female (f) and male (m) yellow mongooses in the Meyersdal estate.
