# Supplementary material for: Flexible Use of Urban Resources by the Yellow Mongoose Cynictis penicillata
Source: Animals (Basel). 2019 Jul 16;9(7):447. doi: 10.3390/ani9070447 (PMC6680935; doi:10.3390/ani9070447)
Supplement: Supplementary file 1 [file animals-09-00447-s001.pdf]

Supplementary material

Flexible use of urban resources by the yellow mongoose, *Cynictis penicillata*.

Submitted to *Animals*

Nadine Elizabeth Cronk, Neville Pillay
Address for correspondence: nadinecronk@gmail.com

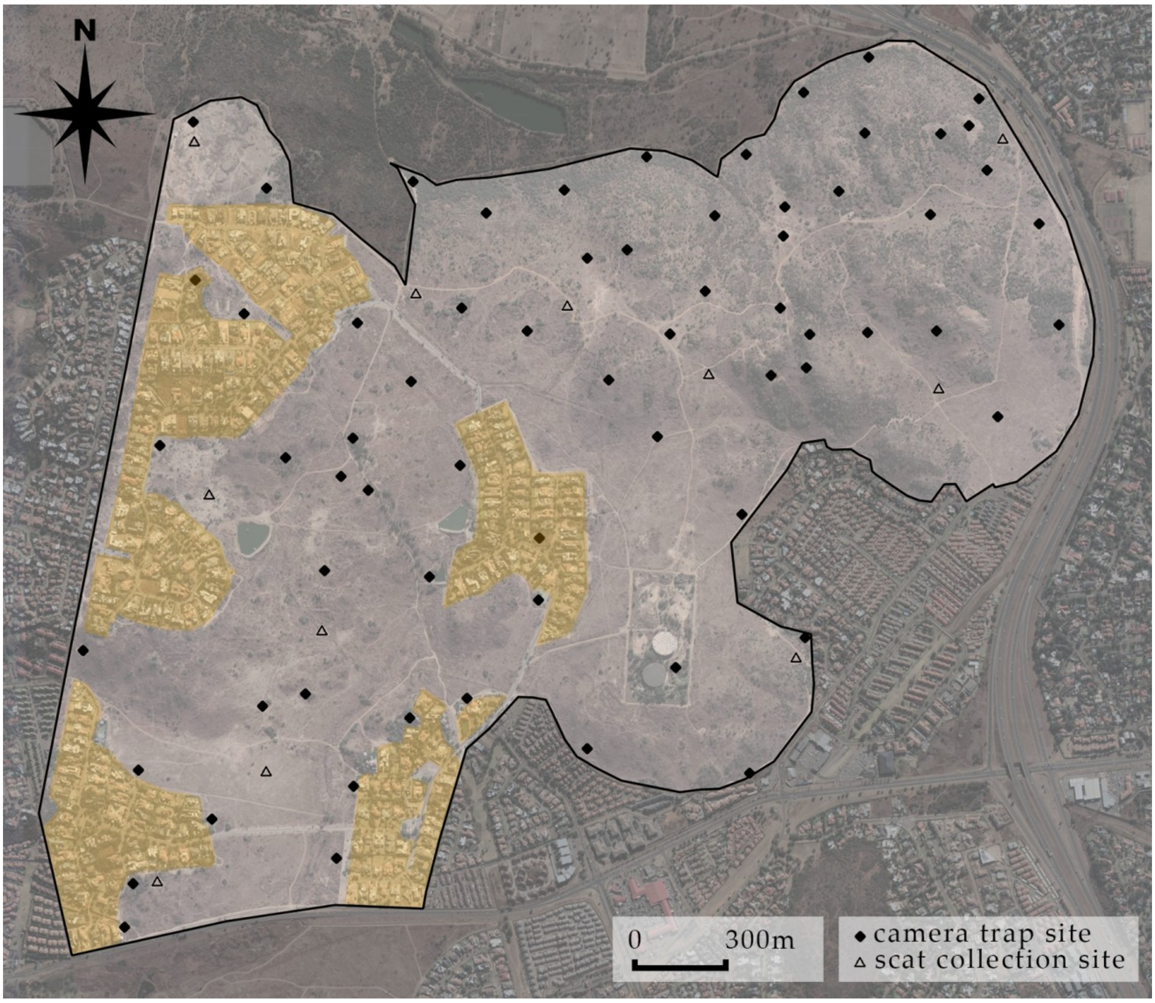

Figure S1. Aerial map of the Meyersdal estate showing the 10 scat sample collection sites (triangles) and the 66 camera trap sites (diamonds); human residences within the estate are highlighted in yellow.

Table S1. Frequency of occurrence of various animal prey remains, identified to appropriate taxonomic levels (order and family), present in a subsample of scats (n=200) of yellow mongooses in the Meyersdal estate.

***Insecta* (*n* = 127)**			***Mammalia* (*n* = 23)**			***Aves* (*n* = 7)**		
**taxa**	***n***	**%**	**taxa**	***n***	**%**	**taxa**	***n***	**%**
Isoptera	89	70.1	Rodentia	19	82.6	Columbidae	6	85.7
Orthoptera	68	53.5	Unidentified	4	17.4	Passeridae	1	14.3
Coleoptera	59	46.5						
Lepidoptera	49	38.6						
Diptera	2	1.57						
Unidentified	11	8.7						

Table S2. Outcomes of the pair-wise comparisons for the LS means of the season and food categories (lsmeans post-hoc analysis output from R statistics).

Season	Contrasts	Estimate	SE	z.ratio	p.value
Autumn	Anthropogenic - Birds	2.01	0.75	2.68	0.037
	Anthropogenic - Insects	-1.39	0.29	-4.80	<0.001
	Anthropogenic - Mammals	0.14	0.38	0.38	0.982
	Birds - Insects	-3.40	0.72	-4.73	<.0001
	Birds - Mammals	-1.87	0.76	-2.46	0.066
	Insects - Mammals	1.53	0.31	4.99	<0.001
Spring	Anthropogenic - Birds	1.87	0.76	2.46	0.066
	Anthropogenic - Insects	-1.68	0.30	-5.58	<.0001
	Anthropogenic - Mammals	0.62	0.47	1.32	0.550
	Birds - Insects	-3.56	0.72	-4.96	<0.001
	Birds - Mammals	-1.25	0.80	-1.56	0.400
	Insects - Mammals	2.30	0.40	5.81	<0.001
Summer	Anthropogenic - Birds	1.79	0.76	2.35	0.088
	Anthropogenic - Insects	-1.81	0.31	-5.80	<0.001
	Anthropogenic - Mammals	0.69	0.50	1.39	0.508
	Birds - Insects	-3.60	0.72	-5.02	<0.001
	Birds - Mammals	-1.10	0.82	-1.35	0.534
	Insects - Mammals	2.50	0.42	5.88	<0.001
Winter	Anthropogenic - Birds	1.53	0.49	3.09	0.011
	Anthropogenic - Insects	-0.58	0.26	-2.22	0.118
	Anthropogenic - Mammals	0.25	0.31	0.78	0.864
	Birds - Insects	-2.10	0.47	-4.44	<0.001
	Birds - Mammals	-1.28	0.51	-2.53	0.055
	Insects - Mammals	0.82	0.28	2.91	0.019

Food	Contrasts	Estimate	SE	z.ratio	p.value
Anthropogenic	Autumns – Spring	1.43	0.38	0.38	0.982
	Autumn – Summer	2.23	0.39	0.58	0.939
	Autumn – Winter	-4.27	0.33	-1.29	0.571
	Spring - Summer	8.00	0.40	0.20	0.997
	Spring – Winter	-571	0.35	-1.64	0.354
	Summer - Winter	-6.51	0.36	-1.83	0.049
Birds	Autumns – Spring	1.47	0.99	0.00	1.000
	Autumn – Summer	5.55	0.99	0.00	1.000
	Autumn – Winter	-9.16	0.84	-1.10	0.693
	Spring - Summer	-9.15	0.99	0.00	1.000
	Spring – Winter	-9.16	0.84	-1.10	0.693
	Summer - Winter	-9.17	0.84	-1.10	0.693
Insects	Autumns – Spring	-1.54	0.18	-0.88	0.817
	Autumn – Summer	-1.96	0.17	-1.3	0.674
	Autumn – Winter	3.81	0.20	1.88	0.237
	Spring - Summer	-4.20	0.17	-0.25	0.995
	Spring – Winter	5.35	0.20	2.72	0.033
	Summer - Winter	5.77	0.19	2.96	0.017
Mammals	Autumns – Spring	6.19	0.47	1.32	0.550
	Autumn – Summer	7.73	0.49	1.57	0.398
	Autumn – Winter	-3.25	0.37	-0.89	0.808
	Spring - Summer	1.54	0.56	0.28	0.993
	Spring – Winter	-9.44	0.45	-2.12	0.147
	Summer - Winter	-1.10	0.50	-2.33	0.091

Table S3. Camera trap site information for the 66 sites sampled within the Meyersdal estate.

Site	Start date	End date	Season	Cover	Distance (m) from human residences	Number of photographs
**1**	08-May-15	21-May-15	Autumn	Open	11.081	104
**2**	08-May-15	21-May-15	Autumn	Closed	1073.627	0
**3**	08-May-15	21-May-15	Autumn	Open	486.52	6
**4**	22-May-15	04-Jun-15	Autumn	Open	25.34	60
**5**	22-May-15	04-Jun-15	Autumn	Closed	976.115	0
**6**	22-May-15	04-Jun-15	Autumn	Closed	263.664	4
**7**	05-Jun-15	18-Jun-15	Winter	Open	37.202	38
**8**	05-Jun-15	18-Jun-15	Winter	Closed	255.768	26
**9**	05-Jun-15	18-Jun-15	Winter	Open	20.001	73
**10**	19-Jun-15	02-Jul-15	Winter	Closed	32.343	21
**11**	19-Jun-15	02-Jul-15	Winter	Closed	434.611	7
**12**	19-Jun-15	02-Jul-15	Winter	Open	23.497	59
**13**	03-Jul-15	16-Jul-15	Winter	Closed	230.815	23
**14**	03-Jul-15	16-Jul-15	Winter	Closed	888.154	0
**15**	03-Jul-15	16-Jul-15	Winter	Open	34.714	56
**16**	17-Jul-15	30-Jul-15	Winter	Closed	125.204	13
**17**	17-Jul-15	30-Jul-15	Winter	Open	419.005	1
**18**	17-Jul-15	30-Jul-15	Winter	Closed	212.234	7
**19**	31-Jul-15	13-Aug-15	Winter	Open	378.938	7
**20**	31-Jul-15	13-Aug-15	Winter	Closed	712.933	1
**21**	31-Jul-15	13-Aug-15	Winter	Open	303.729	10
**22**	14-Aug-15	27-Aug-15	Winter	Open	499.945	4
**23**	14-Aug-15	27-Aug-15	Winter	Closed	1038.928	0
**24**	14-Aug-15	27-Aug-15	Winter	Closed	284.01	7
**25**	28-Aug-15	10-Sep-15	Spring	Open	52.328	37
**26**	28-Aug-15	10-Sep-15	Spring	Closed	699.889	1
**27**	28-Aug-15	10-Sep-15	Spring	Closed	558.362	4
**28**	11-Sep-15	24-Sep-15	Spring	Closed	222.579	11
**29**	11-Sep-15	24-Sep-15	Spring	Closed	827.519	0
**30**	11-Sep-15	24-Sep-15	Spring	Open	259.214	25
**31**	25-Sep-15	08-Oct-15	Spring	Open	331.291	17
**32**	25-Sep-15	08-Oct-15	Spring	Closed	931.99	1
**33**	25-Sep-15	08-Oct-15	Spring	Closed	75.215	25
**34**	09-Oct-15	22-Oct-15	Spring	Open	621.786	25
**35**	09-Oct-15	22-Oct-15	Spring	Closed	301.744	5
**36**	09-Oct-15	22-Oct-15	Spring	Open	61.928	38
**37**	23-Oct-15	05-Nov-15	Spring	Open	23.539	66
**38**	23-Oct-15	05-Nov-15	Spring	Closed	789.602	2
**39**	23-Oct-15	05-Nov-15	Spring	Open	473.659	15
**40**	06-Nov-15	19-Nov-15	Spring	Open	35.054	42
**41**	06-Nov-15	19-Nov-15	Spring	Closed	31.63	23
**42**	06-Nov-15	19-Nov-15	Spring	Closed	701.481	1
**43**	20-Nov-15	03-Dec-15	Spring	Open	492.269	14
**44**	20-Nov-15	03-Dec-15	Spring	Closed	78.136	4
**45**	20-Nov-15	03-Dec-15	Spring	Closed	983.758	0

**46**	04-Dec-15	17-Dec-15	Summer	Open	22.844	75
**47**	04-Dec-15	17-Dec-15	Summer	Open	879.225	1
**48**	04-Dec-15	17-Dec-15	Summer	Closed	44.587	15
**49**	18-Dec-15	31-Dec-15	Summer	Open	25.595	70
**50**	18-Dec-15	31-Dec-15	Summer	Open	666.217	6
**51**	18-Dec-15	31-Dec-15	Summer	Closed	31.503	6
**52**	01-Jan-16	14-Jan-16	Summer	Open	226.898	9
**53**	01-Jan-16	14-Jan-16	Summer	Open	19.549	84
**54**	01-Jan-16	14-Jan-16	Summer	Open	340.134	7
**55**	15-Jan-16	28-Jan-16	Summer	Closed	563.013	8
**56**	15-Jan-16	28-Jan-16	Summer	Open	409.521	4
**57**	15-Jan-16	28-Jan-16	Summer	Closed	294.498	6
**58**	29-Jan-16	11-Feb-16	Summer	Closed	43.331	10
**59**	29-Jan-16	11-Feb-16	Summer	Open	60.008	31
**60**	29-Jan-16	11-Feb-16	Summer	Open	459.574	10
**61**	12-Feb-16	25-Feb-16	Summer	Closed	47.485	12
**62**	12-Feb-16	25-Feb-16	Summer	Closed	500.01	6
**63**	12-Feb-16	25-Feb-16	Summer	Closed	541.595	7
**64**	26-Feb-16	10-Mar-16	Autumn	Open	208.694	32
**65**	26-Feb-16	10-Mar-16	Autumn	Closed	257.32	4
**66**	26-Feb-16	10-Mar-16	Autumn	Open	636.547	3

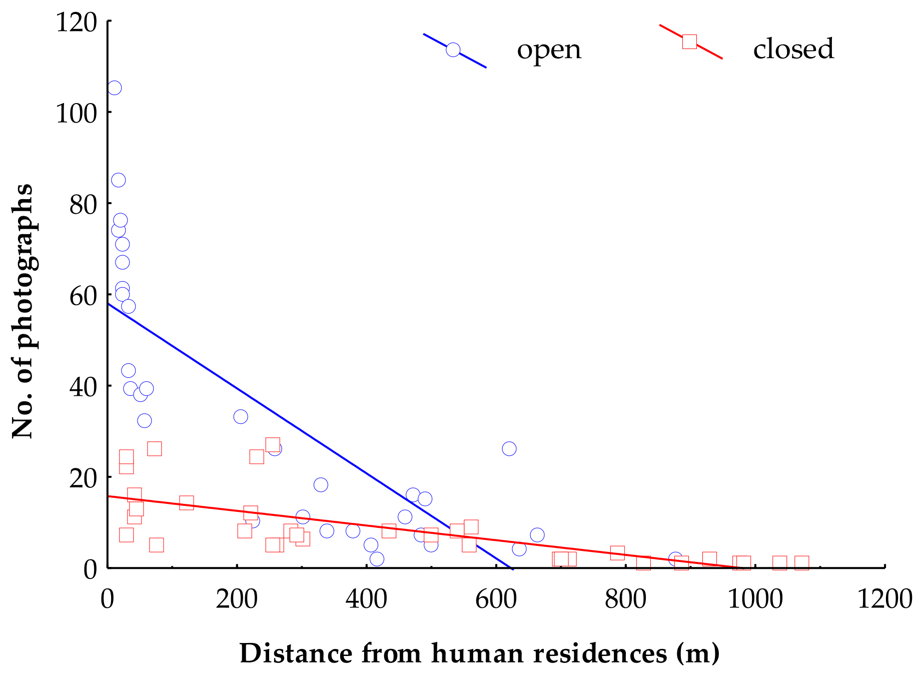

Figure S2. The number of photographs of yellow mongooses captured on camera traps at different distances from human residences in open or closed vegetation cover in the Meyersdal estate.

Table S4. Comparison of male and female yellow mongoose home range sizes (mean ±SE; range), based on two estimates (MCP and KDE) for the 50%, 95% and 100% isopleth level in the Meyersdal estate. No estimates were possible for the KDE 100% isopleth

Method	Mean (±SE; range) home range size	
	**Female**	**Male**
MCP 50%	1.72 (0.09; 0.18)	4.38 (1.61; 3.22)
MCP 95%	7.16 (0.26; 0.52)	14.61 (4.48; 8.96)
MCP 100%	8.74 (0.18; 0.36)	21.13 (4.96; 9.92)
KDE 50%	1.56 (0.13; 0.26)	3.54 (1.22; 2.44)
KDE 95%	6.50 (0.27; 0.53)	13.37 (4.61; 9.22)

Table S5. Seasonal home range sizes, based on two estimates (MCP and KDE), of female (f) and male (m) yellow mongooses in the Meyersdal estate.

Name	Season	MCP			KDE	
		50%	95%	100%	50%	95%
**m1**	Autumn	1.86	8.50	9.04	1.24	6.49
	Winter	0.94	4.49	6.63	0.42	4.84
**f1**	Autumn	1.43	5.06	5.67	1.31	4.87
	Winter	0.67	3.11	3.69	0.63	3.22
**m2**	Spring	3.02	12.15	14.57	2.33	10.19
	Summer	3.57	13.90	14.33	3.39	11.67
**f2**	Autumn	2.11	7.32	8.30	1.85	6.44
	Spring	0.52	1.76	2.29	0.52	1.59
	Summer	1.23	2.47	2.89	0.59	2.55

Table S6. Seasonal percentage of total GPS locations (n = number of GPS locations) overlapping with human residential gardens, of female (f) and male (m) yellow mongooses in the Meyersdal estate.

Name	Season	% GPS location overlap (n)		
		**50%**	**95%**	**100%**
**m1**	Autumn	5.24 (11)	26.19 (55)	29.05 (61)
	Winter	21.35 (19)	34.83 (31)	37.08 (33)
**f1**	Autumn	0.00 (0)	8.06 (17)	11.37 (24)
	Winter	0.00 (0)	9.18 (9)	16.33 (16)
**m2**	Spring	0.00 (0)	21.37 (56)	23.28 (61)
	Summer	3.28 (6)	14.21 (26)	17.49 (32)
**f2**	Autumn	3.83 (7)	14.75 (27)	18.03 (33)
	Spring	0.00 (0)	5.41 (2)	10.81 (4)
	Summer	0.75 (0)	1.13 (3)	2.26 (6)
